# Effects of Chinese Herbal Medicines on the Risk of Overall Mortality, Readmission, and Reoperation in Hip Fracture Patients

**DOI:** 10.3389/fphar.2019.00629

**Published:** 2019-06-11

**Authors:** Chi-Fung Cheng, Ying-Ju Lin, Fuu-Jen Tsai, Te-Mao Li, Ting-Hsu Lin, Chiu-Chu Liao, Shao-Mei Huang, Xiang Liu, Ming-Ju Li, Bo Ban, Wen-Miin Liang, Jeff Chien-Fu Lin

**Affiliations:** ^1^Genetic Center, Department of Medical Research, China Medical University Hospital, Taichung, Taiwan; ^2^Graduate Institute of Biostatistics, School of Public Health, China Medical University, Taichung, Taiwan; ^3^School of Chinese Medicine, China Medical University, Taichung, Taiwan; ^4^Department of Biotechnology and Bioinformatics, Asia University, Taichung, Taiwan; ^5^National Institute of Allergy and Infectious Diseases, National Institutes of Health, Bethesda, MD, United States; ^6^Chinese Research Center for Behavior Medicine in Growth and Development, Jining, China; ^7^Department of Statistics, National Taipei University, Taipei, Taiwan; ^8^Department of Orthopedic Surgery, Wan Fang Hospital, Taipei Medical University, Taipei, Taiwan

**Keywords:** hip fracture, Chinese herbal medicine, overall mortality, readmission, reoperation

## Abstract

Hip fracture is a major public health concern, with high incidence rates in the elderly worldwide. Hip fractures are associated with increased medical costs, patient dependency on families, and higher rates of morbidity and mortality. Chinese herbal medicine (CHM) is typically characterized as cost-effective and suitable for long-term use with few side effects. To better understand the effects of CHM on hip fracture patients, we utilized a population-based database to investigate the demographic characteristics, cumulative incidence of overall mortality, readmission, reoperation, and patterns of CHM prescription. We found that CHM usage was associated with a lower risk of overall mortality [*P* = 0.0009; adjusted hazard ratio (HR): 0.47, 95% confidence interval (CI): 0.30–0.73], readmission (*P* = 0.0345; adjusted HR: 0.67, 95% CI: 0.46–0.97), and reoperation (*P* = 0.0009; adjusted HR: 0.57, 95% CI: 0.40–0.79) after adjustment for age, type of hip fracture, surgical treatment type, and comorbidities. We also identified the herbal formulas, single herbs, and prescription patterns for the treatment of hip fracture by using association rule mining and network analysis. For hip fracture patients, the most common CHM coprescription pattern was Du-Zhong (DZ) → Xu-Duan (XD), followed by Du-Huo-Ji-Sheng-Tang (DHJST) → Shu-Jing-Huo-Xue-Tang (SJHXT), and Gu-Sui-Bu (GSB) → Xu-Duan (XD). Furthermore, XD was the core prescription, and DZ, GSB, SJHXT, and DHJST were important prescriptions located in cluster 1 of the prescription patterns. This study provides evidence for clinical CHM use as an adjunctive therapy that offers benefits to hip fracture patients.

## Introduction

Hip fracture is a major public health concern with a high incidence rate, especially in elder patients worldwide (Friedman and Mendelson, 2014; Lin and Liang, 2017). An estimated 6.26 million hip fracture patients will exist worldwide by 2050 (Gullberg et al., 1997). Half of these, about 2.5 million hip fractures, will occur in Asia (Dhanwal et al., 2011). In Taiwan, among the elderly, hip fracture patients increased from 3% of the population in 1964 to 10.7% in 2011 (Wang et al., 2013). Patients with hip fractures incur increased costs of medical care, increased dependency on families, and have higher morbidity and mortality outcomes. Surgery, including hemiarthroplasty and internal fixation of fractures, is frequently used for the management of hip fractures. However, patient outcomes of morbidity and mortality and their relationships to current treatments require further scrutiny (Wang et al., 2013; Lin and Liang, 2017). To reduce the incidence of hip fracture and to reduce the outcomes of overall mortality, readmission, and reoperation of hip fracture patients, numerous approaches have been proposed and pursued including improved osteoporosis screening, diagnosis and medications, fracture prevention programs, and research-supported integrative, alternative, and complementary nutrition and medicine.

Chinese herbal medicine (CHM) is typically characterized as cost-effective, suitable for long-term use, and associated with relatively few side effects. It has been extensively used as a complementary therapy for the treatment of many diseases and ailments in Taiwan (Shih et al., 2012; Liao et al., 2015; Tsai et al., 2017a; Li et al., 2018a; Tsai et al., 2018). CHM has also been used to treat bone-related diseases including osteoporosis and bone fractures (Shih et al., 2012; Mukwaya et al., 2014; Liao et al., 2015). CHM is believed to maintain bone health, including: inhibition of inflammation, promotion of fracture healing, osteopenia prevention, and antiosteoporotic activities (Chow et al., 1982; Chen et al., 2005; Li et al., 2011; Ma et al., 2011; Xiang et al., 2011; Wong et al., 2013; He and Shen, 2014; Zhang et al., 2016; Hsiao et al., 2017; Wang et al., 2018c; Xi et al., 2018; Lee et al., 2019). These studies have encouraged the search for complementary therapy for the better management of bone-related diseases. As such, an investigation into the clinical use of CHM in combination with regular therapy in hip fracture patients is appropriate and necessary.

To better understand the incidence and effects of CHM as treatment in hip fracture patients, we utilized a population-based database to investigate the demographic characteristics, cumulative incidence of overall mortality, readmission, reoperation, and patterns of CHM prescription for hip fracture patients. Through this retrospective population-based case–control analysis, we were able to investigate whether the use of CHM as adjunctive therapy offers benefits to hip fracture patients.

## Materials and Methods

### Data Source

To examine whether CHM use is associated with a lower risk of overall mortality, readmission, and reoperation after hip fracture, a population-based retrospective cohort study was conducted. Subjects were identified based on the International Classification of Disease, 9th Revision, Clinical Modification (ICD-9-CM). This population was part of a database comprising all individuals 40 years of age or older who received surgery for hip fracture based on a) first discharge disease codes of hip fracture: ICD-9-CM: 820, 820.0, 820.00, 820.01, 820.02, 820.09, 820.8, 820.03, 820.2, 820.20, and 820.21; and b) procedure codes with surgery of internal fixation or hemiarthroplasty (based on ICD-9-CM: 79.15, 79.35, and 81.52) during the period from 2000 to 2010 who were included in the National Health Insurance Research database (NHIRD; http://nhird.nhri.org.tw/) of the National Health Insurance (NHI) program. This program includes the total population of patients in Taiwan (23 million individuals) and includes 99% of the general population; it is only used for research purposes by scientists in Taiwan. All personal data were decoded for identity, so we were unable to obtain an informed consent. This database provides detailed medical records including information on age, gender, diagnoses, prescriptions, records of clinical visits and hospitalizations, inpatient orders, ambulatory care, and sociodemographic factors. This database also offers longitudinally linked data for the period from 1996 to 2012. The study was approved by the Institutional Review Board of China Medical University Hospital.

### Subjects

**Figure 1:**
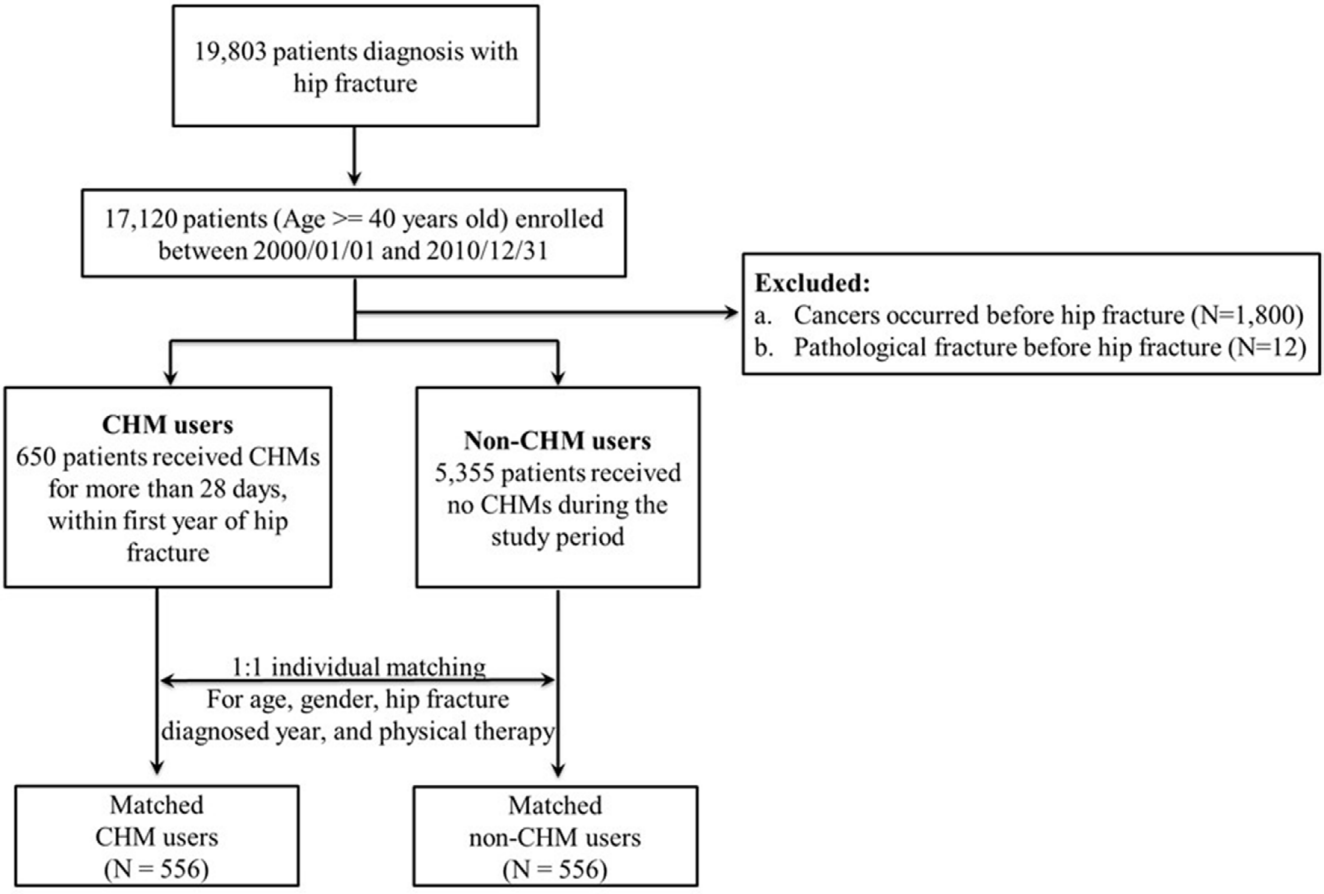
Flowchart used for identification and enrollment of study subjects.

The first admission date due to a hip fracture was defined as the diagnostic day of the hip fracture. The exclusion criteria included subjects with cancers (ICD-9-CM 140–172, 174–195.8, and 200–208), which occurred before hip fracture or those with pathological fractures (ICD-9-CM: 733.14 and 733.15) before hip fracture. Subjects who underwent surgery for injuries to the pelvis, femur, or hip region before the index day were also excluded to avoid confounding effects. Individuals with more than 28 cumulative CHM treatment days within the first year after a diagnosis of hip fracture were defined as CHM users (*n* = 650, **Figure 1**). The study subjects who did not receive any CHM were defined as nonusers of CHM (*n* = 5,355). In addition, to reduce bias due to confounding variables, nonusers were selected at a 1:1 ratio with CHM users *via* individual matching for age, gender, year of hip fracture diagnosis, and physical therapy. In total, 556 and 556 subjects were selected as CHM and nonusers, respectively (**Figure 1** and **Table 1**). The day on which the 28 cumulative days within 1 year of CHM treatment were completed was designated as the index date. In this study, distribution of the cumulative period of CHM treatment of CHM users within 365 days after the index date is shown in **Table S5**. The study endpoint for overall mortality was defined as the date of death, the date of withdrawal from the NHI program, or the date of termination of follow-up (December 31, 2012) (**Tables S3** and **S4**).

**Table 1:** Demographic characteristics of total subjects and individual matched subjects of hip fracture patients.

Characteristics	Total subjects			Individual matched subjects		
	CHM users	Non-CHM users	*P* value	CHM users	Non-CHM users	*P* value
	*n* = 650	*n* = 5,355		*n* = 556	*n* = 556	
	*n* (%)	*n* (%)		*n* (%)	*n* (%)	
**Age**			***<0.001***			0.385
<60 years old	129 (19.85%)	626 (11.69%)		82 (14.75%)	72 (12.95%)	
≥60 years old	521 (80.15%)	4,729 (88.31%)		474 (85.25%)	484 (87.05%)	
**Gender**			***<0.001***			1.000
Male	273 (42%)	2,634 (49.19%)		240 (43.17%)	240 (43.17%)	
Female	377 (58%)	2,721 (50.81%)		316 (56.83%)	316 (56.83%)	
**Physical therapy**			***<0.001***			1.000
No	577 (88.77%)	4,979 (92.98%)		515 (92.63%)	515 (92.63%)	
Yes	73 (11.23%)	376 (7.02%)		41 (7.37%)	41 (7.37%)	
**Type of hip fracture**			***<0.001***			0.717
Intertrochanter fracture of femur	277 (42.62%)	2,709 (50.59%)		244 (43.88%)	250 (44.96%)	
Intracapsular fracture of the femoral neck	373 (57.38%)	2,646 (49.41%)		312 (56.12%)	306 (55.04%)	
**Surgery type of hip fracture**			0.808			0.853
Hemiarthroplasty	239 (36.77%)	1,943 (36.28%)		211 (37.95%)	208 (37.41%)	
Internal fixation of fracture	411 (63.23%)	3,412 (63.72%)		345 (62.05%)	348 (62.59%)	
**Comorbidities**						
Hypertension	276 (42.46%)	2,522 (47.1%)	***0.025***	248 (44.60%)	271 (48.74%)	0.167
Diabetes	144 (22.15%)	1,299 (24.26%)	0.236	128 (23.02%)	150 (26.98%)	0.128
Heart diseases	85 (13.08%)	994 (18.56%)	***<0.001***	78 (14.03%)	95 (17.09%)	0.160
Chronic obstructive pulmonary disease	82 (12.62%)	909 (16.97%)	***0.005***	74 (13.31%)	95 (17.09%)	0.079
Cerebrovascular diseases	83 (12.77%)	1,123 (20.97%)	***<0.001***	71 (12.77%)	93 (16.73%)	0.062
Chronic liver diseases	30 (4.62%)	276 (5.15%)	0.555	24 (4.32%)	35 (6.29%)	0.141
Chronic renal diseases	17 (2.62%)	273 (5.1%)	***0.005***	16 (2.88%)	25 (4.50%)	0.152

n, number; CHM, Chinese herbal medicine.

Age, gender, physical therapy, type of hip fracture, surgery type of hip fracture, and comorbidities were expressed as categorical variable [number (%)].

P values were obtained by chi-square test. Significant P values (P < 0.05) were highlighted in bold italic.

The study endpoint for readmission was defined as the date of the first medical readmission due to medical complications within 365 days after index date. Readmission within 365 days after index date may be caused directly or indirectly by the surgery itself. Readmission included medical complications occurring within 365 days after which extra days of hospital stay or readmission to the hospital was required for additional treatment including stroke, acute myocardial infarction, pulmonary embolism, acute renal failure, or acute respiratory failure.

The study endpoint for reoperation was defined as the date of the first reoperation due to surgical complications within 365 days after index date. Reoperation included conversion to or revision of an arthroplasty, surgical site infection, removal of an implant due to complications, mechanical complications (including loss reduction, screw back-out or cut-out, skin irritation, and implant failure), dislocation, avascular necrosis of the femoral head, second hip fracture, and malunion/nonunion during the follow-up period.

The patient demographic characteristics are shown in **Table 1**, including age, gender, physical therapy, type of hip fracture, surgery type of hip fracture treatment, and comorbidities. We identified comorbidities that had been diagnosed in the study subjects before or at the time of the index day, including hypertension (ICD-9-CM 401–405), diabetes (ICD-9-CM 250.0–250.3, and 250.7), heart diseases (ICD-9-CM 410–414), chronic obstructive pulmonary disease (ICD-9-CM 490–496), cerebrovascular diseases (ICD-9-CM 430–438), chronic liver diseases (ICD-9-CM 571.2, 571.4–571.6, 070.4, 070.5, and 070.7), and chronic renal diseases (ICD-9-CM 582, 583–583.7, 585, 586, and 588).

### Chinese Herbal Medicine

There are two kinds of Chinese herbal medicine (CHM) products: herbal formulas and single herbs. Herbal formulas are composed of a combination of two or more herbs provided by knowledgeable traditional Chinese medicine (TCM) practitioners based on TCM or ancient medical books (**Table S1** and **Table S2**). Single herbs may be from plants, animals, or mineral sources. The codes for herbal formulas and single herbs were collected, grouped, and listed on the Taiwan NHI website (http://www.nhi.gov.tw/webdata/webdata.aspx?menu=21&menu_id=713&webdata_id=932). These CHM products in Taiwan are personally prescribed to patients for many kinds of ailments by experienced TCM doctors and are all manufactured by pharmaceutical manufacturers with Good Manufacturing Practice certifications. The main pharmaceutical manufacturers are Sun Ten Pharmaceutical Co. Ltd. (http://www.sunten.com.tw/), Chuang Song Zong Pharmaceutical Co. Ltd. (http://www.csz.com.tw/), Shang Chang Pharmaceutical Co. Ltd. (http://www.herb.com.tw/about_en.php), KO DA Pharmaceutical Co. Ltd. (http://www.koda.com.tw/), and Kaiser Pharmaceutical Co. Ltd (http://www.kpc.com/). For CHM products, the frequency of prescriptions, frequency of users, person-years, percentage of people using that CHM, average drug dose (per day), and average duration of the prescription were calculated from the index date to the study end (**Table S1**).

### Statistical Analysis

**Figure 2:**
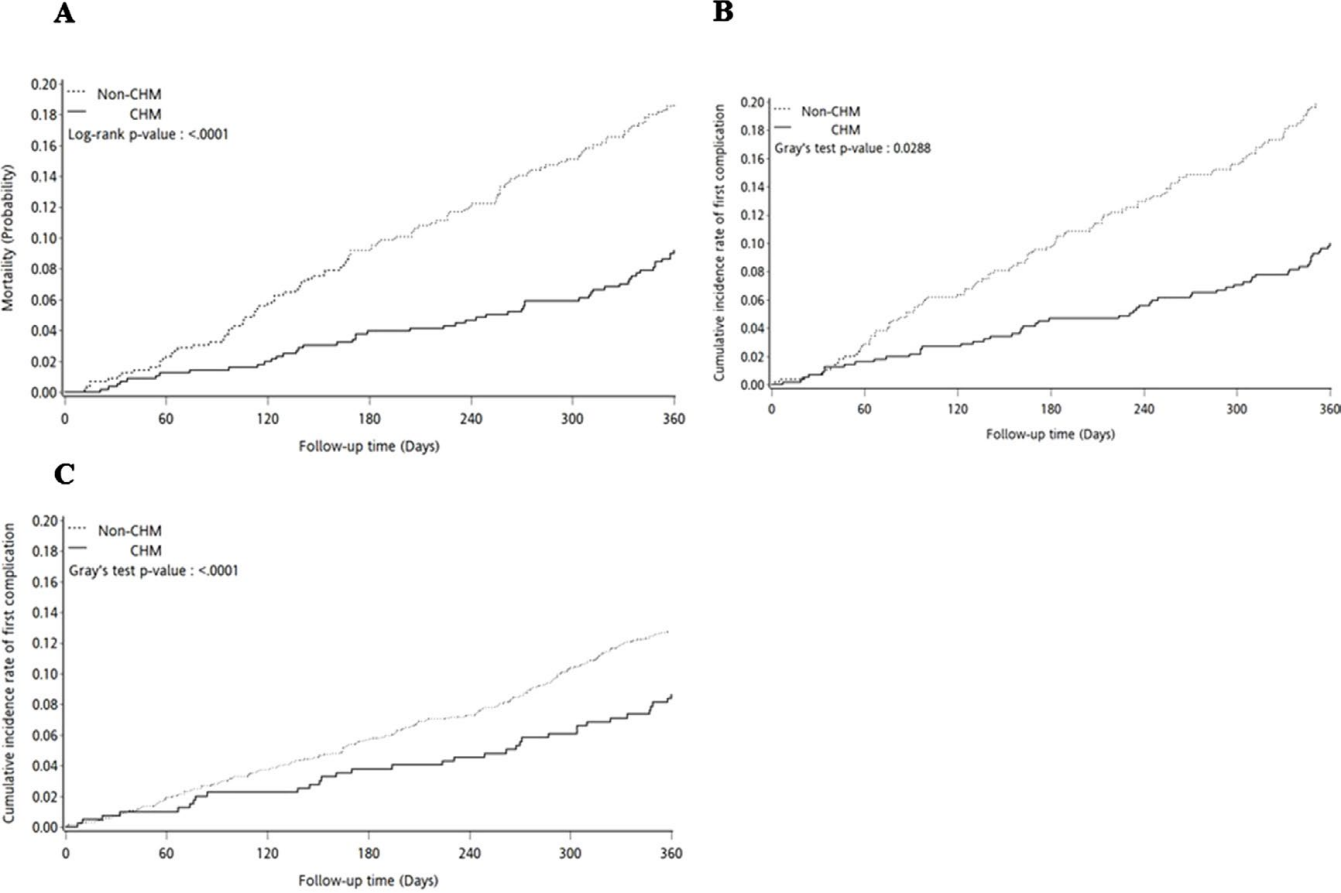
Comparison of the cumulative incidence between Chinese herbal medicine (CHM) and non-CHM users in hip fracture patients. **(A)** Cumulative incidence of the overall mortality. **(B)** Cumulative incidence of readmission. **(C)** Cumulative incidence of reoperation.

**Figure 3:**
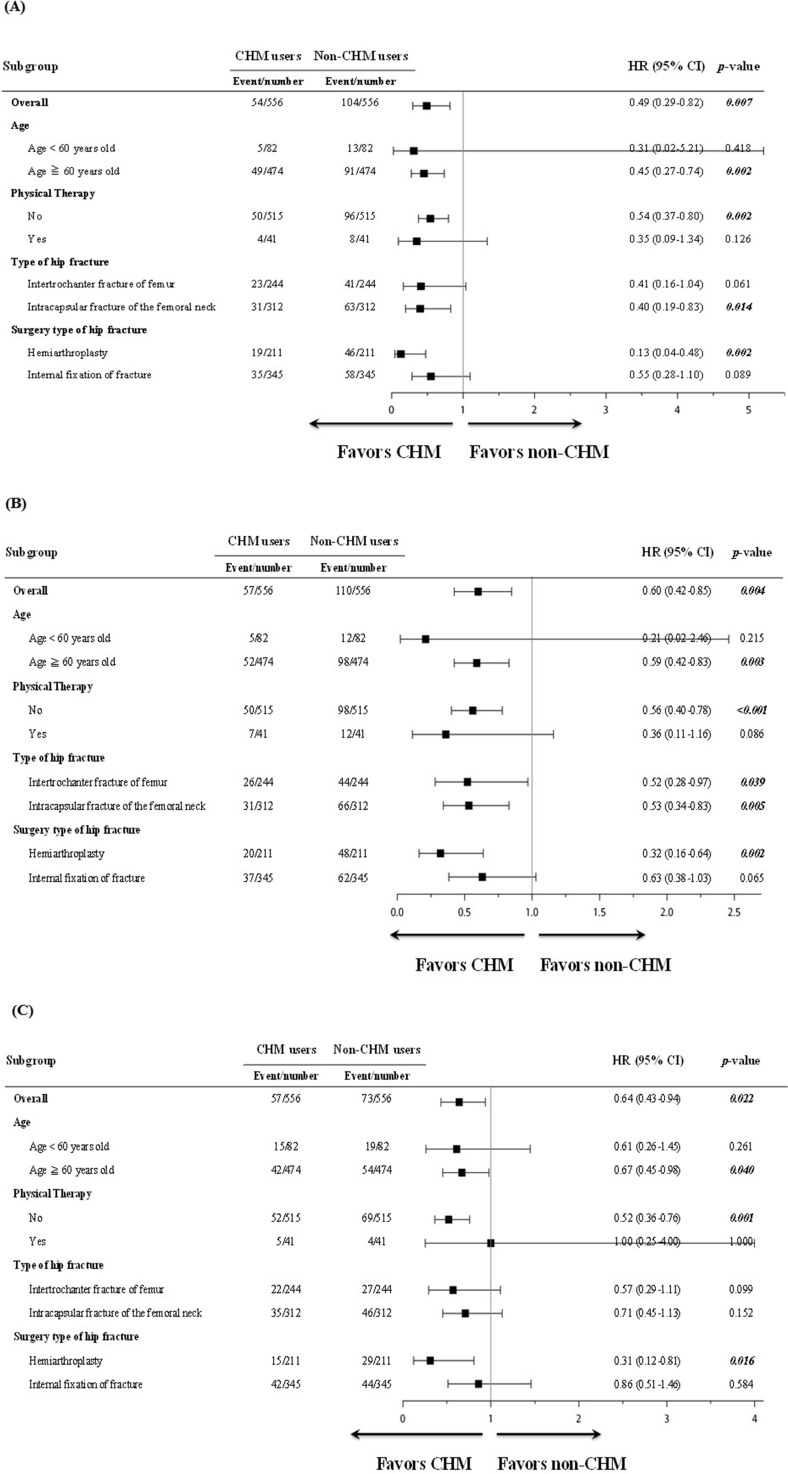
Subgroup analysis for the risk of overall mortality, readmission, and reoperation in hip fracture patients. **(A)** Hazard ratios (HRs) and 95% confidence intervals (CI) for overall mortality were adjusted for confounding factors and stratified by age, physical therapy, type of hip fracture, and surgery type. The event and total number of each subgroup between CHM and non-CHM users are also shown. **(B)** HRs and 95% CI for readmission were adjusted for confounding factors and stratified by age, physical therapy, type of hip fracture, and surgery type. The event and total number of each subgroup between CHM and non-CHM users are also shown. **(C)** HRs and 95% CI for reoperation were adjusted for confounding factors and stratified by age, physical therapy, type of hip fracture, and surgery type of hip fracture. The event and total number of each subgroup between CHM and non-CHM users are also shown.

Categorical data are expressed as numbers and percentages. These include age, gender, physical therapy, type of hip fracture, surgery type, and comorbidities including hypertension, diabetes, heart diseases, chronic obstructive pulmonary disease, cerebrovascular diseases, chronic liver diseases, and chronic renal diseases (**Table 1**). The significance of the differences of the categorical data was calculated using a chi-squared test (**Table 1**). A Cox proportional hazard model was applied to assess the hazard ratio (HR) of mortality for CHM users when compared with nonusers with adjustment for age, type of hip fracture, surgery type of hip fracture, and comorbidities (**Table 2**). Furthermore, a Fine and Gray’s hazard model was performed to assess the hazard ratio (HR) of the risks of readmission and reoperation for CHM users when compared with nonusers with adjustment for age, type of hip fracture, surgery type of hip fracture, and comorbidities (**Tables 3** and **4**). The frequency and usage patterns of the 10 most common herbal formulas and single herbs used are shown in **Table S1**. Coprescriptions of single herbs and herbal formulas for hip fracture patients were shown by using the association rules (Yang et al., 2013) (**Table 5**). Association rule mining was computed using the ‘‘arules_1.6’’ package of the R software (version 3.4.3). The Kaplan–Meier method, the log-rank test, and the Gray’s test were performed to estimate the 365-day cumulative incidence of mortality, readmission, and reoperation according to CHM use (**Figure 2A–C**). Furthermore, for the risk of overall mortality, hip fracture patients were stratified according to age, physical therapy, type of hip fracture, and surgery type (**Figure 3A**). For the risk of readmission, the hip fracture patients were stratified according to age, physical therapy, type of hip fracture, and surgery type (**Figure 3B**). For the risk of reoperation, the hip fracture patients were stratified according to age, physical therapy, type of hip fracture, and surgery type (**Figure 3C**). The network analysis using Cytoscape (http://manual.cytoscape.org/en/stable/Network_Analyzer.html) was applied to explore the CHM network and the core treatments for these hip fracture patients from the NHIRD database in Taiwan. The red color indicates the herbal formula, and the green color indicates a single herb. The size of the circle represents the user number of each CHM. Larger circles mean higher frequencies of user numbers. The connection between CHMs represents user numbers for the CHM combinations. A more important connection between CHMs is indicated by a thicker and darker connection line. All *P* values less than 0.05 were considered to be statistically significant. All data management and statistical analyses were performed using SAS software (version 9.4; Statistical Analysis Software [SAS] Institute, Cary, NC, USA).

**Table 2:** Cox proportional hazard models for overall mortality of hip fracture patients.

Variable	Number of deaths (*n* = 158)	Number of hip fracture patients (*n* = 1,112)	Crude			Adjusted		
	*n* (%)	*n* (%)	HR	(95% CI)	*P* value	aHR	(95% CI)	*P* value
**Age**	ND	ND	1.03	(1.01–1.04)	0.0003	1.09	(0.97–1.24)	0.1549
**CHM use**								
Yes	54 (9.7%)	556 (100.0%)	0.44	(0.30–0.63)	***<0.0001***	0.47	(0.30–0.73)	***0.0009***
No	104 (18.7%)	556 (100.0%)	1.00	Reference		1.00	Reference	
**Type of hip fracture**								
Intracapsular fracture of the femoral neck	80 (12.9%)	618 (100.0%)	0.80	(0.59–1.09)	0.1578	1.83	(0.77–4.37)	0.1723
Intertrochanter fracture of femur	78 (15.8%)	494 (100.0%)	1.00	Reference		1.00	Reference	
**Surgery type of hip fracture**								
Internal fixation of fracture	107 (15.4%)	693 (100.0%)	1.30	(0.93–1.81)	0.1244	2.47	(1.01–6.05)	***0.0475***
Hemiarthroplasty	51 (12.2%)	419 (100.0%)	1.00	Reference		1.00	Reference	
**Comorbidities**								
Hypertension								
Yes	92 (17.7%)	519 (100.0%)	1.64	(1.20–2.25)	***0.0021***	1.43	(0.74–2.75)	0.2877
No	66 (11.1%)	593 (100.0%)	1.00	Reference		1.00	Reference	
Diabetes								
Yes	51 (18.4%)	278 (100.0%)	1.48	(1.06–2.07)	***0.0206***	1.66	(0.81–3.39)	0.1672
No	107 (12.8%)	834 (100.0%)	1.00	Reference		1.00	Reference	
Heart diseases								
Yes	34 (19.7%)	173 (100.0%)	1.55	(1.06–2.26)	***0.0244***	0.67	(0.33–1.37)	0.2722
No	124 (13.2%)	939 (100.0%)	1.00	Reference		1.00	Reference	
COPD								
Yes	34 (20.1%)	169 (100.0%)	1.63	(1.12–2.38)	***0.0117***	0.70	(0.34–1.46)	0.3435
No	124 (13.2%)	943 (100.0%)	1.00	Reference		1.00	Reference	
Cerebrovascular diseases								
Yes	33 (17.6%)	188 (100.0%)	1.34	(0.91–1.97)	0.1358	1.26	(0.54–2.92)	0.5935
No	125 (13.5%)	924 (100.0%)	1.00	Reference		1.00	Reference	
Chronic liver diseases								
Yes	8 (13.6%)	59 (100.0%)	0.96	(0.47–1.95)	0.9059	0.91	(0.24–3.44)	0.8879
No	150 (14.3%)	1,053 (100.0%)	1.00	Reference		1.00	Reference	
Chronic renal diseases								
Yes	9 (22.0%)	41 (100.0%)	1.68	(0.86–3.29)	0.1319	1.39	(0.29–6.60)	0.6783
No	149 (13.9%)	1,071 (100.0%)	1.00	Reference		1.00	Reference	

HR, hazard ratio; 95% CI, 95% confidence interval; ND, not determined; COPD, chronic obstructive pulmonary disease; aHR, adjusted hazard ratio.

Age (years) was expressed as a continuous variable. Therefore, the number (%) for age was not determined. The risk of overall mortality increased with age (HR 1.09/year) in our study.

Models adjusted for age, CHM use, type of hip fracture, surgery type of hip fracture, and comorbidities.

p value < 0.05 shown in bold italic.

**Table 3:** Fine and Gray’s hazard models for readmission risk in hip fracture patients.

Variable	Number of readmission (*n* = 158)	Number of hip fracture patients (*n* = 1,112)	Crude			Adjusted		
	*n* (%)	*n* (%)	HR	(95% CI)	*P* value	aHR	(95% CI)	*P* value
**Age**	ND	ND	1.02	(1.01–1.03)	***0.0013***	0.98	(0.89–1.09)	0.6984
**CHM use**								
Yes	110 (19.8%)	556 (100.0%)	0.49	(0.38–0.60)	***<0.0001***	0.67	(0.46–0.97)	***0.0345***
No	57 (10.3%)	556 (100.0%)	1.00	Reference		1.00	Reference	
**Type of hip fracture**								
Intracapsular fracture of the femoral neck	78 (15.8%)	494 (100.0%)	0.90	(0.66–1.21)	0.4748	2.34	(1.23–4.43)	***0.0094***
Intertrochanter fracture of femur	89 (14.4%)	618 (100.0%)	1.00	Reference		1.00	Reference	
**Surgery type of hip fracture**								
Internal fixation of fracture	61 (14.6%)	419 (100.0%)	1.07	(0.78–1.46)	0.6938	2.38	(1.13–5.01)	***0.0228***
Hemiarthroplasty	106 (15.3%)	693 (100.0%)	1.00	Reference		1.00	Reference	
**Comorbidities**								
Hypertension								
Yes	59 (10.0%)	593 (100.0%)	2.21	(1.61–3.04)	***<0.0001***	1.19	(0.72–1.95)	0.4957
No	108 (20.8%)	519 (100.0%)	1.00	Reference		1.00	Reference	
Diabetes								
Yes	110 (13.2%)	834 (100.0%)	1.61	(1.17–2.21)	***0.0034***	1.51	(0.88–2.60)	0.1322
No	57 (20.5%)	278 (100.0%)	1.00	Reference		1.00	Reference	
Heart diseases								
Yes	133 (14.2%)	939 (100.0%)	1.44	(0.99–2.10)	0.0590	0.54	(0.25–1.13)	0.1017
No	34 (19.7%)	173 (100.0%)	1.00	Reference		1.00	Reference	
COPD								
Yes	131 (13.9%)	943 (100.0%)	1.62	(1.12–2.35)	***0.0106***	0.92	(0.44–1.91)	0.8206
No	36 (21.3%)	169 (100.0%)	1.00	Reference		1.00	Reference	
Cerebrovascular diseases								
Yes	114 (12.3%)	924 (100.0%)	2.58	(1.85–3.58)	***<0.0001***	0.88	(0.40–1.97)	0.7636
No	53 (28.2%)	188 (100.0%)	1.00	Reference		1.00	Reference	
Chronic liver diseases								
Yes	160 (15.2%)	1,053 (100.0%)	0.77	(0.36–1.66)	0.5093	0.42	(0.13–1.32)	0.1377
No	7 (11.9%)	59 (100.0%)	1.00	Reference		1.00	Reference	
Chronic renal diseases								
Yes	160 (14.9%)	1,071 (100.0%)	1.19	(0.55–2.60)	0.6558	0.78	(0.17–3.57)	0.7476
No	7 (17.1%)	41 (100.0%)	1.00	Reference		1.00	Reference	

Age (years) was expressed as a continuous variable. Therefore, the number (%) for age was not determined.

Models adjusted for age, CHM use, type of hip fracture, surgery type of hip fracture, and comorbidities.

Death was used as a competing risk.

p value < 0.05 shown in bold italic.

**Table 4:** Fine and Gray’s hazard models for reoperation risk in hip fracture patients.

Variable	Number of reoperation (*n* = 158)	Number of hip fracture patients (*n* = 1,112)	Crude			Adjusted		
	*n* (%)	*n* (%)	HR	(95% CI)	*P* value	aHR	(95% CI)	*P* value
**Age**	ND	ND	0.98	(0.97–1.00)	***0.0046***	1.07	(0.97–1.17)	0.1620
**CHM use**								
Yes	73 (13.1%)	556 (100.0%)	0.74	(0.56–0.97)	***0.0304***	0.57	(0.40–0.79)	***0.0009***
No	57 (10.3%)	556 (100.0%)	1.00	Reference		1.00	Reference	
**Type of hip fracture**								
Intracapsular fracture of the femoral neck	53 (10.7%)	494 (100.0%)	1.17	(0.83–1.66)	0.3715	1.93	(1.01–3.66)	***0.0456***
Intertrochanter fracture of femur	77 (12.5%)	618 (100.0%)	1.00	Reference		1.00	Reference	
**Surgery type of hip fracture**								
Internal fixation of fracture	39 (9.3%)	419 (100.0%)	1.45	(1.00–2.10)	0.0531	2.31	(1.20–4.44)	***0.0118***
Hemiarthroplasty	91 (13.1%)	693 (100.0%)	1.00	Reference		1.00	Reference	
**Comorbidities**								
Hypertension								
Yes	55 (9.3%)	593 (100.0%)	1.59	(1.12–2.25)	***0.0092***	1.57	(0.96–2.59)	0.0753
No	75 (14.5%)	519 (100.0%)	1.00	Reference		1.00	Reference	
Diabetes								
Yes	87 (10.4%)	834 (100.0%)	1.51	(1.05–2.18)	***0.0256***	2.11	(1.20–3.73)	***0.0098***
No	43 (15.5%)	278 (100.0%)	1.00	Reference		1.00	Reference	
Heart diseases								
Yes	111 (11.8%)	939 (100.0%)	0.91	(0.56–1.46)	0.6833	0.58	(0.33–1.02)	0.0577
No	19 (11.0%)	173 (100.0%)	1.00	Reference		1.00	Reference	
COPD								
Yes	115 (12.2%)	943 (100.0%)	0.72	(0.42–1.23)	0.2280	0.86	(0.48–1.52)	0.5955
No	15 (8.9%)	169 (100.0%)	1.00	Reference		1.00	Reference	
Cerebrovascular diseases								
Yes	106 (11.5%)	924 (100.0%)	1.12	(0.72–1.73)	0.6287	2.58	(1.41–4.69)	***0.0020***
No	24 (12.8%)	188 (100.0%)	1.00	Reference		1.00	Reference	
Chronic liver diseases								
Yes	125 (11.9%)	1,053 (100.0%)	0.69	(0.29–1.65)	0.4007	0.97	(0.41–2.28)	0.9363
No	5 (8.5%)	59 (100.0%)	1.00	Reference		1.00	Reference	
Chronic renal diseases								
Yes	126 (11.8%)	1,071 (100.0%)	0.82	(0.30–2.20)	0.6885	0.47	(0.18–1.28)	0.1409
No	4 (9.8%)	41 (100.0%)	1.00	Reference		1.00	Reference	

Age (years) was expressed as a continuous variable. Therefore, the number (%) for age was not determined.

Models adjusted for age, CHM use, type of hip fracture, surgery type of hip fracture, and comorbidities.

Death was used as a competing risk.

p value < 0.05 shown in bold italic.

**Table 5:** Ten most commonly used pairs of CHM products for hip fracture patients in Taiwan.

CHM products (LHS, X)	Chinese name		CHM products (RHS, Y)	Chinese name	Frequency of prescriptions of X and Y products	Support (X) (%)	Confidence (X → Y) (%)	Lift
Du-Zhong (DZ)	杜仲	→	Xu-Duan (XD)	續斷	388	2.5	39.8	6.3
Du-Huo-Ji-Sheng-Tang (DHJST)	獨活寄生湯	→	Shu-Jing-Huo-Xue-Tang (SJHXT)	疏經活血湯	372	2.4	24.1	2.2
Gu-Sui-Bu (GSB)	骨碎補	→	Xu-Duan (XD)	續斷	330	2.1	38.2	6.0
Xu-Duan (XD)	續斷	→	Shu-Jing-Huo-Xue-Tang (SJHXT)	疏經活血湯	221	1.4	21.9	2.0
Xu-Duan (XD)	續斷	→	Du-Huo-Ji-Sheng-Tang (DHJST)	獨活寄生湯	215	1.4	21.4	2.2
Da-Huang (DH)	大黃	→	Dan-Shen (DS)	丹參	213	1.4	21.2	2.9
Xue-Fu-Zhu-Yu-Tang (XFZYT)	血府逐瘀湯	→	Dan-Shen (DS)	丹參	208	1.3	29.0	4.0
Gu-Sui-Bu (GSB)	骨碎補	→	Du-Huo-Ji-Sheng-Tang (DHJST)	獨活寄生湯	208	1.3	24.0	2.5
Yan-Hu-Suo (YHS)	延胡索	→	Shu-Jing-Huo-Xue-Tang (SJHXT)	疏經活血湯	202	1.3	17.5	1.6
Du-Zhong (DZ)	杜仲	→	Du-Huo-Ji-Sheng-Tang (DHJST)	獨活寄生湯	200	1.3	20.5	2.1

CHM, Chinese herbal medicine; LHS, left-hand side; RHS, right-hand side.

Support (X) (%) = Frequency of prescriptions of X and Y products/total prescriptions × 100%.

Confidence (X → Y) (%) = Frequency of prescriptions of X and Y products/Frequency of prescriptions of X product × 100%.

P (Y) (%) = Frequency of prescriptions of Y product/total prescriptions × 100%.

Lift = Confidence (X → Y) (%)/P (Y) (%).

## Results

### Demographic Characteristics of Study Patients

Overall, 19,803 hip fractures were diagnosed between 2000 and 2010 (**Figure 1**). Of these, 17,120 hip fracture patients 40 years of age or older were enrolled between 2000 and 2010. Patients were further excluded due to cancers that occurred before the hip fracture (*n* = 1,800) and pathological fracture before the hip fracture (*n* = 12). These exclusions left 650 patients assigned to the CHM user group and 5,355 patients regarded as nonusers who did not use CHMs during the study period. As shown in **Table 1**, there were differences in age, gender, physical therapy, type of hip fracture, and comorbidities (hypertension, heart disease, chronic obstructive pulmonary disease, cerebrovascular diseases, and chronic renal diseases) between these two groups (total subjects; *P* < 0.05; **Table 1**). After individual matching of subjects in the CHM user group and nonuser group for age, gender, hip fracture diagnosed years, and physical therapy, 556 and 556 patients were included in the two groups, respectively (**Figure 1**). There were no significant differences between the two matched groups (**Table 1**; *P* > 0.05).

### Cumulative Incidence and Cox Proportional Hazard of Overall Mortality Between Chinese Herbal Medicine and Non-Chinese Herbal Medicine Users in Hip Fracture Patients in Taiwan

The 365-day cumulative incidence of overall mortality was shown using the Kaplan–Meier survival curve (**Figure 2A**). A difference was identified in the probability of overall mortality between these two groups (log-rank test, *P* < 0.0001). The cumulative incidence of overall mortality was significantly lower in CHM users than in nonusers. A multivariate Cox proportional hazard model was performed to estimate the hazard ratio (HR) and 95% confidence interval (CI) of overall mortality associated with the CHM users and covariates among hip fracture patients. Compared with hip fracture patients who did not receive CHM treatment, those who did had a lower risk of overall mortality after adjustment for age, type of hip fracture, surgery type, and comorbidities (aHR: 0.47, 95% CI: 0.30–0.73, *P* = 0.0009; **Table 2**). Compared with hip fracture patients who had hemiarthroplasty surgery, those who had internal fixation of fracture surgery had a higher risk of overall mortality (aHR: 2.47, 95% CI: 1.01–6.05, *P* = 0.0475; **Table 2**).

The HRs for overall mortalities of these hip fracture patients following division into subgroups according to age, physical therapy, type of hip fracture, and surgery type of hip fracture are shown (**Figure 3A**). Among these subgroups, the HRs for overall mortality risk among CHM users were lower than those of non-CHM users. Subgroup analysis showed that the use of CHM was associated with a protective effect in those who were aged 60 years or older (HR: 0.45, 95% CI: 0.27–0.74, *P* = 0.002), in those without physical therapy (HR: 0.54, 95% CI: 0.37–0.80, *P* = 0.002), in those with intracapsular fracture of the femoral neck (HR: 0.40, 95% CI: 0.19–0.83, *P* = 0.014), and in those who had hemiarthroplasty surgery (HR: 0.13, 95% CI: 0.04–0.48, *P* = 0.002).

### Cumulative Incidence and Fine and Gray’s Hazard for Readmission Risk Between Chinese Herbal Medicine and Non- Chinese Herbal Medicine Users in Hip Fracture Patients in Taiwan

The 365-day cumulative incidence of readmission was illustrated by the Kaplan–Meier survival curve (**Figure 2B**). The readmission outcome was observed by using death as the competing risk. The cumulative incidence of readmission was significantly lower in CHM users than in nonusers (Readmission: Gray’s test, *P* = 0.0288). A multivariate Fine and Gray’s proportional hazard model was also applied to estimate the hazard ratio (HR) and 95% confidence interval (CI) of readmission associated with the CHM users and covariates among the hip fracture patients using death as the competing risk (**Table 3**). Compared with the hip fracture patients who did not receive CHM treatment, CHM users had a lower risk of readmission than nonusers after adjustment for age, type of hip fracture, surgery type, and comorbidities (aHR: 0.67, 95% CI: 0.46–0.97, *P* = 0.0345; **Table 3**). Compared with the hip fracture patients who had intertrochanter fracture of the femur, patients who had an intracapsular fracture of the femoral neck had a higher risk of readmission (aHR: 2.34, 95% CI: 1.23–4.43, *P* = 0.0094; **Table 3**). Compared with the hip fracture patients who had hemiarthroplasty surgery, patients who underwent internal fixation had a higher risk of readmission (aHR: 2.38, 95% CI: 1.13–5.01, *P* = 0.0228; **Table 3**).

The HRs for readmission of these hip fracture patients following division into subgroups according to age, physical therapy, type of hip fracture, and surgery type are shown (**Figure 3B**). Among these subgroups, the HRs for the risk of readmission among CHM users were lower than those of non-CHM users. Subgroup analysis for the HR for readmission showed that use of CHM was associated with a protective effect in those who were aged 60 years or older (HR: 0.59, 95% CI: 0.42–0.83, *P* = 0.003), in those without physical therapy (HR: 0.56, 95% CI: 0.40–0.78, *P* < 0.001), in both types of hip fracture (HR: 0.52, 95% CI: 0.28–0.97, *P* = 0.039 and HR: 0.53, 95% CI: 0.34–0.83, *P* = 0.005, respectively), and in those who had hemiarthroplasty surgery (HR: 0.32, 95% CI: 0.16–0.64, *P* = 0.002) (**Figure 3B**).

### Cumulative Incidence and Fine and Gray’s Hazard for Reoperation Risk Between Chinese Herbal Medicine and Non- Chinese Herbal Medicine Users in Hip Fracture Patients in Taiwan

The 365-day cumulative incidence of reoperation was illustrated by the Kaplan–Meier survival curve (**Figure 2C**). The reoperation outcome was observed by using death as the competing risk. The cumulative incidence of reoperation was significantly lower in CHM users than in nonusers (Reoperation: Gray’s test, *P* < 0.0001). A multivariate Fine and Gray’s proportional hazard model was also applied to estimate the hazard ratio (HR) and 95% confidence interval (CI) of reoperation associated with the CHM users and covariates among the hip fracture patients using death as the competing risk (**Table 4**). Compared with the hip fracture patients who did not receive CHM treatment, CHM users had a lower risk of reoperation than nonusers after adjustment for age, type of hip fracture, surgery type, and comorbidities (aHR: 0.57, 95% CI: 0.40–0.79, *P* = 0.0009; **Table 4**). Compared with the hip fracture patients who had intertrochanter fracture of femur, the patients who had intracapsular fracture of the femoral neck had a higher risk of reoperation (aHR: 1.93, 95% CI: 1.01–3.66, *P* = 0.0456; **Table 4**). Compared with the hip fracture patients who had hemiarthroplasty surgery, the patients who underwent internal fixation had a higher risk of reoperation (aHR: 2.31, 95% CI: 1.20–4.44, *P* = 0.0118; **Table 4**). There were significantly higher risks of reoperation among the hip fracture patients who had comorbidities such as diabetes (aHR: 2.11, 95% CI: 1.20–3.73, *P* = 0.0098; **Table 4**) and cerebrovascular diseases (aHR: 2.58, 95% CI: 1.41–4.69, *P* = 0.0020; **Table 4**).

The HRs for reoperation of these hip fracture patients following division into subgroups according to age, physical therapy, type of hip fracture, and surgery type are shown (**Figure 3C**). Of these subgroups, the HRs for the risk of reoperation among CHM users were lower than those of non-CHM users. Subgroup analysis for the HR for reoperation showed that use of CHM was associated with a protective effect in those who were aged 60 years or older (HR: 0.67, 95% CI: 0.45–0.98, *P* = 0.040), in those without physical therapy (HR: 0.52, 95% CI: 0.36–0.76, *P* = 0.001), and in those who had hemiarthroplasty surgery (HR: 0.31, 95% CI: 0.12–0.81, *P* = 0.016) (**Figure 3C**).

### Most Commonly Prescribed Chinese Herbal Formulas and Single Herbs by Traditional Chinese Medicine Doctors for the Treatment of Hip Fracture Patients

The 10 most commonly prescribed herbal formulas and 10 single herbs used for the treatment of hip fracture patients are listed (**Table S1**). The compositions of these CHM products are also presented (**Table S2**). According to the frequency of prescription, Shu-Jing-Huo-Xue-Tang (SJHXT) (40.8%) was the most commonly prescribed herbal formula. The second and third most common formulas were Du-Huo-Ji-Sheng-Tang (DHJST) (37.2%) and Ma-Zi-Ren-Wan (MZRW) (25.2%). Yan-Hu-Suo (YHS) [*Corydalis yanhusuo* (Y.H. Chou and Chun C. Hsu) W.T. Wang ex Z.Y. Su and C.Y. Wu, 36.2%] was the most commonly prescribed single herb, followed by Dan-Shen (DS) (*Salvia miltiorrhiza* Bunge, 31.1%) and Niu-Xi (NX) (*Achyranthes bidentata* Blume, 35.6%).

The coprescription patterns of the most commonly used CHM products were also studied in hip fracture patients by using association rules (**Table 5**). The support (%), confidence (%), and lift of the association rules of these 10 most commonly used pairs were explored. The coprescription patterns with higher values of support, confidence, and lift were more strongly correlated in hip fracture patients. As shown in **Table 5**, for hip fracture patients, the CHM coprescription pattern (Du-Zhong (DZ) → Xu-Duan (XD); support: 2.5%, confidence: 39.8%, lift: 6.3) had the highest value of support data, which suggested that this coprescription pattern had the most significant association for the treatment of hip fracture, followed by Du-Huo-Ji-Sheng-Tang (DHJST) → Shu-Jing-Huo-Xue-Tang (SJHXT) (second coprescription; support: 2.4%, confidence: 24.1%, lift: 2.2) and Gu-Sui-Bu (GSB) → Xu-Duan (XD) (third coprescription; support: 2.1%, confidence: 38.2%, lift: 6.0).

**Figure 4:**
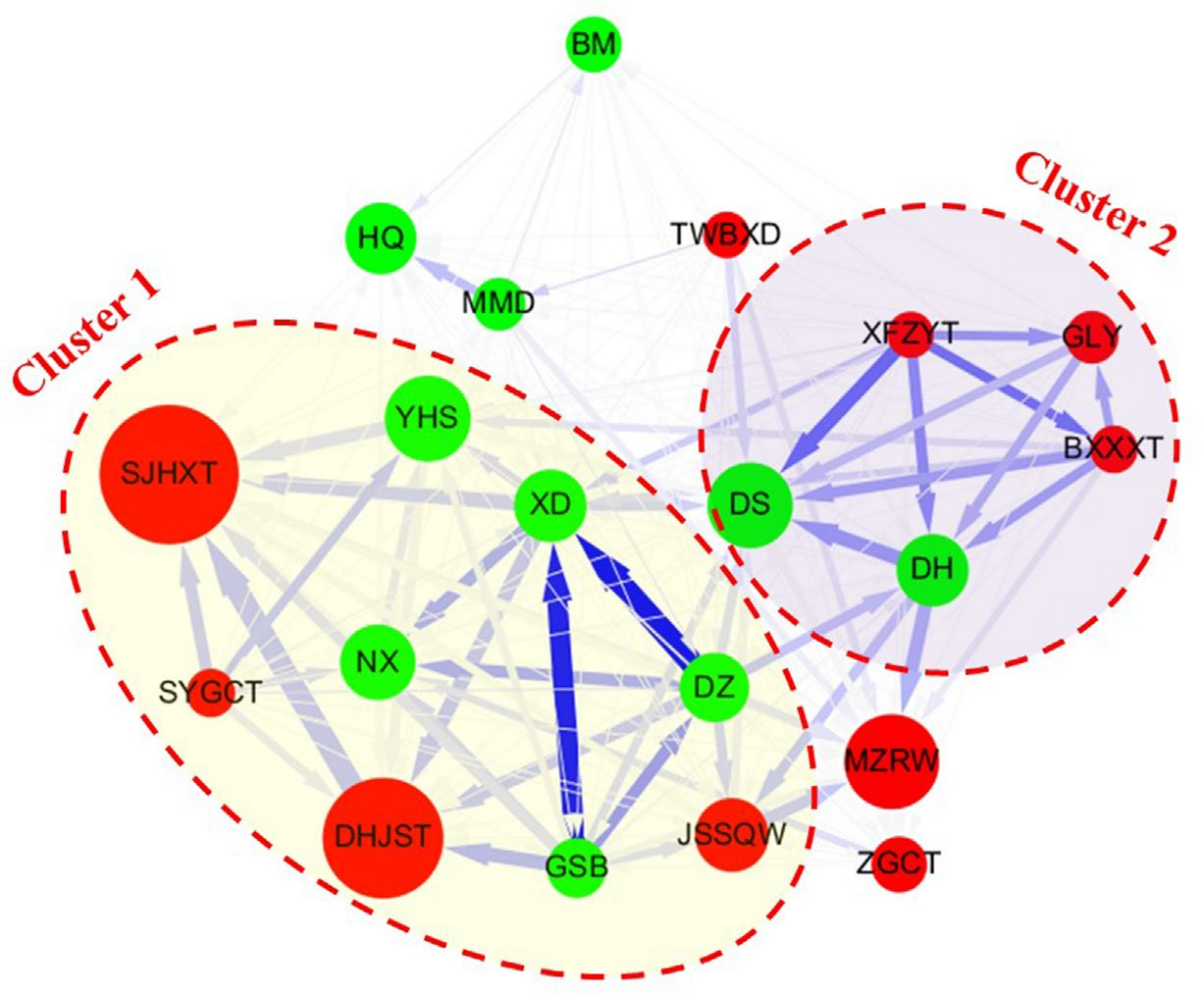
The CHM network for hip fracture patients. The red circle represents herbal formulas; the green circle represents single herbs. The size of the circle represents the frequency of prescription for each CHM. A larger circle represents a higher frequency of prescription. The lines connecting the CHMs represent the confidence value for the different CHM combinations. The thicker line means a higher value of confidence. The blue line color represents the life value for the different CHM combinations. The darker blue color means a higher value of lift

To further explore the CHM network for hip fracture patients, their coprescription patterns and networks were identified. These networks highlight the complicated relationships among the CHM products (**Figure 4**). There were 556 hip fracture patients who used CHM products and 20,326 prescriptions were provided by TCM doctors (**Table 4**). In addition, two clusters were identified by the association rule and network analysis (**Table 5** and **Figure 4**). Cluster 1 was the largest CHM cluster, and the major CHM in this cluster was different compared with cluster 2. In cluster 1, XD was the core CHM, and DZ, GSB, SJHXT, and DHJST were important CHMs. Among cluster 1, DZ, XD, and GSB had significant associations with each other according to the support, confidence, and lift values (DZ → XD: support: 2.5%, confidence: 39.8%, lift: 6.3; GSB → XD: support: 2.1%, confidence: 38.2%, lift: 6.0) (**Table 5** and **Figure 4**). In cluster 2, XFZYT was the core CHM, and DS, DH, GLY, and BXXXT were important CHMs. Among cluster 2, DH, DS, and XFZYT had significant associations with each other according to the support, confidence, and lift values (DH → DS: support: 1.4%, confidence: 21.2%, lift: 2.9; XFZYT → DS: support: 1.3%, confidence: 29.0%, lift: 4.0) (**Table 5** and **Figure 4**).

### Discussion

In this retrospective, population-based, case–control study, we investigated the demographic characteristics, cumulative incidence of overall mortality, readmission, reoperation, and patterns of CHM prescription in hip fracture patients in Taiwan. We found that CHM usage was associated with lower risks of overall mortality, readmission, and reoperation after adjustment for age, type of hip fracture, surgery type, and comorbidities. We also identified the herbal formulas, single herbs, and prescription patterns for the treatment of hip fracture by using association rule mining. Therefore, this study provides evidence of clinical CHM use as adjunctive therapy benefiting hip fracture patients.

We recruited hip fracture patients, 40 years of age or older, who underwent surgeries for hip fracture. Notably, about 85% of these patients were more than 60 years of age and about 56% were female. The risk of hip fracture is greater in postmenopausal women and seniors and is probably related to osteoporosis (Metcalfe, 2008). Osteoporosis is one of the most common types of bone diseases, resulting from an imbalance between bone formation and resorption (Infante and Rodriguez, 2018). It is characterized by a degeneration of the bone microstructure, reduction of bone mass, and higher fracture risks. As CHM is cost-effective with relatively few side effects and has been widely applied for clinical use in Asian countries, it has been previously used for the clinical treatment of osteoporosis and bone fracture in Taiwan (Shih et al., 2012; Liao et al., 2015). Indeed, there are several Chinese herbs that help maintain bone health by regulating bone metabolism (Chow et al., 1982; Chen et al., 2005; Li et al., 2011; Ma et al., 2011; Xiang et al., 2011; Wong et al., 2013; He and Shen, 2014; Zhang et al., 2016; Hsiao et al., 2017; Wang et al., 2018c; Xi et al., 2018). Our pharmacoepidemiologic results have demonstrated that for the patients who were above 60 years old, there was a significant distribution difference in the cumulative overall mortality between CHM and non-CHM users (**Table S3** and **Table S4**). Our results showed the protective effects of clinically used CHM on mortality and outcomes after surgeries in hip fracture patients.

Among the most commonly used pairs of CHM products for hip fracture patients, the CHM coprescription pattern Du-Zhong → Xu-Duan (support: 2.5) resulted in the highest support, followed by Du-Huo-Ji-Sheng-Tang → Shu-Jing-Huo-Xue-Tang (second coprescription; support: 2.4), and Gu-Sui-Bu → Xu-Duan (third coprescription; support: 2.1). Du-Zhong (DZ; *Eucommiae cortex*) is the dried trunk bark of *Eucommia ulmoides* Oliv., of the Eucommiaceae family. Du-Zhong (DZ) has been used for the treatment of fractures, osteoporosis, and rheumatoid arthritis (Shih et al., 2012; Gao et al., 2013; Liao et al., 2015; Wu et al., 2017; Qi et al., 2018; Wang et al., 2018a). Studies have reported that extracts of Du-Zhong exhibit anti-inflammatory, antitumor, collagen synthesizing, and antiosteoporotic properties (Li et al., 2000; Ha et al., 2003; Kim et al., 2012; Kang et al., 2013; Tan et al., 2014; Li et al., 2016; Wang et al., 2016a; Zhou et al., 2016; Koh et al., 2017). Natural compounds of Du-Zhong, including 5-(hydroxymethyl)-2-furaldehyde and chlorogenic acid, show antiosteoporotic activity *via* promoting osteoblast-like cell proliferation and osteoclast inhibition (Tan et al., 2014; Zhou et al., 2016).

Xu-Duan (XD; *Radix Dipsaci*) is the dried root of *Dipsacus asperoides* C.Y. Cheng and T.M.Ai of the Teasel family. Xu-Duan (XD) has been used for the treatment of fractures, osteoporosis, and rheumatoid arthritis (Liu et al., 2009; Peng et al., 2010; Jung et al., 2012; Liu et al., 2012; Shih et al., 2012; Liao et al., 2015; Ke et al., 2016; Li et al., 2016). Treatment of Xu-Duan extracts have exhibited anti-inflammatory, antiarthritic, and antiosteoporotic activities (Wong et al., 2007; Liu et al., 2009; Kim et al., 2011; Jung et al., 2012; Niu et al., 2015a). Natural compounds of Xu-Duan, including asperosaponin VI and saponins, are involved in bone metabolism (Niu et al., 2012; Niu et al., 2015b; Ke et al., 2016). Asperosaponin VI promotes osteogenic differentiation through the phosphoinositide-3-kinase/AKT serine/threonine kinase (PI3K/AKT) signaling pathway in bone marrow stromal cells (Ke et al., 2016). Saponins from Xu-Duan exert an effect on osteoblastic maturation and differentiation through the bone morphogenetic protein (BMP)-2/mitogen-activated protein kinase/Smad1/5/8-dependent Runx2 signaling pathways in MC3T3-E1 mouse osteoblast precursor cells (Niu et al., 2015b).

Gu-Sui-Bu (GSB; *Drynariae rhizoma*) is the dried rhizome of *Drynaria fortunei* (Kunze ex Mett). J.Sm. of the Polypodiaceae family. Gu-Sui-Bu (GSB) has been used for the treatment of fractures, osteoporosis, rheumatoid arthritis, and head injuries (Shih et al., 2012; Wang et al., 2012; Saravanan et al., 2013; Liao et al., 2015). Studies have reported that extracts of Gu-Sui-Bu exhibit immune-promoting, anti-inflammatory, antiosteoporotic, and neuroprotective activities (Anuja et al., 2010; Chen et al., 2011b; Wang et al., 2012; Saravanan et al., 2013; Kang et al., 2014; Wang et al., 2016b). The natural compounds of Gu-Sui-Bu include naringin and flavonoids (Wang et al., 2008; Chen et al., 2011b). Naringin from Gu-Sui-Bu increases the proliferation and differentiation of MC3T3-E1 osteoblastic cells (Chen et al., 2011b). Flavonoids from Gu-Sui-Bu show proliferative activity in UMR106 osteoblast-like cells (Wang et al., 2008).

Du-Huo-Ji-Sheng-Tang (DHJST) is composed of 15 single herbs. DHJST has been used for the treatment of fractures, osteoporosis, osteoarthritis, aging in the elderly, rheumatoid arthritis, and stroke in type 2 diabetes (Chen et al., 2011a; Shih et al., 2012; Chen et al., 2014; Liao et al., 2015; Yang et al., 2015; Chen et al., 2016; Tsai et al., 2017a; Wang et al., 2017). Studies have reported that DHJST extracts promote osteogenic differentiation, antiaging, anti-inflammatory activities, and therapeutic effects in osteoarthritis (Chen et al., 2011a; Yang et al., 2015; Chen et al., 2016; Wang et al., 2017). The natural compound *Ligusticum chuanxiong* from DHJST increases osteogenic activity in human mesenchymal stem cells by up-regulating BMP-2 and RUNX2 expression *via* SMAD 1/5/8 and ERK signaling and also delays the cell aging process by decreasing cell senescence in human mesenchymal stem cells (Wang et al., 2017).

Shu-Jing-Huo-Xue-Tang (SJHXT) is composed of 17 single herbs. SJHXT has been used for the treatment of fractures, osteoporosis, adjuvant arthritis, prostate cancer, breast cancer, hypertension, and type 2 diabetes (Kanai et al., 2003; Shu et al., 2010; Lin et al., 2012; Tsai et al., 2014; Liao et al., 2015; Tsai et al., 2017a, Tsai et al., 2017b, Tsai et al., 2017c). SJHXT extracts showed antihypersensitivity and pain relief effects by increasing blood circulation (Kanai et al., 2003; Shu et al., 2010). The natural compounds constituting SJHXT include ferulic acid and paeoniflorin. Ferulic acid promotes osteogenesis in bone marrow mesenchymal stem cells and suppresses osteoclast differentiation (Du et al., 2017; Doss et al., 2018). Paeoniflorin has a significant anti-inflammatory effect on rheumatoid arthritis (Lai et al., 2018; Xu et al., 2018). Paeoniflorin also shows antiosteoporosis activity and regulates osteoclastogenesis and osteoblastogenesis (Li and Chent 2018b; Wang et al., 2018b).

In conclusion, this study demonstrated that the CHM users had lower hazard ratios for the risk of overall mortality, readmission, and reoperation when compared with CHM nonusers among hip fracture patients. Based on association rule mining, Du-Zhong → Xu-Duan were most strongly associated with each other for the specific treatment of hip fractures. The use of CHM as an adjunctive therapy may reduce the risks of overall mortality, readmission, and reoperation; therefore, further clinical and experimental studies should be performed to optimize the safety and efficacy of CHM use in these patients.

## Ethics Statement

This database also offers longitudinally linked data for the period from 1996 to 2012. All personal data were decoded for identity, so we were unable to obtain informed consent. The study was approved by the Institutional Review Board of China Medical University Hospital.

## Author Contributions

C-FC, JC-FL, F-JT, W-ML, and Y-JL conceived and designed the experiments. C-FC, T-HL, C-CL, and S-MH performed the experiments. C-FC and M-JL analyzed the data. T-ML, XL, BB, and Y-JL contributed with reagents/materials/analysis tools. W-ML and Y-JL wrote the manuscript. All of the authors have read and approved the final manuscript.

## Funding

This study was supported by grants from China Medical University (CMU107-S-13 and CMU107-S-15), China Medical University Hospital (DMR-107-042, DMR-108-113, DMR-108-114, and DMR-108-118), and the National Science Council, the Ministry of Science and Technology, Taiwan (MOST 105-2314-B-039-037-MY3, MOST 106-2320-B-039-017-MY3).

## Conflict of Interest Statement

The authors declare that the research was conducted in the absence of any commercial or financial relationships that could be construed as a potential conflict of interest.
