# Supplementary material for: Effects of Chinese Herbal Medicines on the Risk of Overall Mortality, Readmission, and Reoperation in Hip Fracture Patients
Source: Front Pharmacol. 2019 Jun 11;10:629. doi: 10.3389/fphar.2019.00629 (PMC6581068; doi:10.3389/fphar.2019.00629)
Supplement: Supplementary file 1 [file DataSheet_1.docx]

**Supplementary Material**

**Table S1.** The ten most common herbal formulas and single herbs for hip fracture patients in Taiwan.

**Table S2.** Composition of the most commonly used herbal formulas and single herbs for hip fracture patients in Taiwan.

**Table S3.** Cumulative overall mortality rate of hip fracture patients stratified by age and follow-up time (within 365 days after the index date).

**Table S4.** Cumulative overall mortality rate of hip fracture patients stratified by age and follow-up time (within 12 years after the index date).

**Table S5.** Distribution of the cumulative period of CHM treatment of CHM users among hip fracture patients in this study in Taiwan (within 365 days after the index date).

| **TABLE S1 \|** The ten most common herbal formulas and single herbs for hip fracture patients in Taiwan. | | | | | | | |
| --- | --- | --- | --- | --- | --- | --- | --- |
| **Formulas** | **Chinese name** | **Frequency of prescriptions** | **Frequency of user** | **Person-year** | **Percentage of usage person** | **Avg. drug dose per day (g)** | **Average duration for prescription (days)** |
| **Total** |  | 20326 | 556 | 2883.8 | 100.0 | 12.4 | 7.9 |
| **Herbal formula (Pin-yin name) (Shorten name)** |  | 19524 | 555 | 2881.5 | 99.8 | 9.4 | 7.9 |
| **Shu-Jing-Huo-Xue-Tang (SJHXT)** | 疏經活血湯 | 1767 | 227 | 1384.6 | 40.8 | 4.3 | 8.3 |
| **Du-Huo-Ji-Sheng-Tang (DHJST)** | 獨活寄生湯 | 1554 | 207 | 1282.5 | 37.2 | 5.0 | 8.0 |
| **Ma-Zi-Ren-Wan (MZRW)** | 麻子仁丸 | 1259 | 140 | 719.7 | 25.2 | 3.0 | 9.8 |
| **Ji-Sheng-Shen-Qi-Wan (JSSQW)** | 濟生腎氣丸 | 1026 | 159 | 863.8 | 28.6 | 4.5 | 8.2 |
| **Zhi-Gan-Cao-Tang (ZGCT)** | 炙甘草湯 | 827 | 130 | 725.2 | 23.4 | 4.4 | 8.2 |
| **Gan-Lu-Yin (GLY)** | 甘露飲 | 794 | 129 | 815.4 | 23.2 | 3.9 | 7.2 |
| **Shao-Yao-Gan-Cao-Tang (SYGCT)** | 芍藥甘草湯 | 741 | 183 | 1075.1 | 32.9 | 3.3 | 7.1 |
| **Ban-Xia-Xie-Xin-Tang (BXXXT)** | 半夏瀉心湯 | 733 | 110 | 592.6 | 19.8 | 3.6 | 8.6 |
| **Xue-Fu-Zhu-Yu-Tang (XFZYT)** | 血府逐瘀湯 | 724 | 147 | 867.5 | 26.4 | 3.9 | 8.7 |
| **Tian-Wang-Bu-Xin-Dan (TWBXD)** | 天王補心丹 | 723 | 125 | 717.2 | 22.5 | 4.1 | 8.1 |
| **Single herbs (Pin-yin name)** |  | 16279 | 536 | 2787.8 | 96.4 | 4.1 | 8.1 |
| **Yan-Hu-Suo (YHS)** | 延胡索 | 1169 | 201 | 1190.2 | 36.2 | 1.1 | 7.7 |
| **Dan-Shen (DS)** | 丹參 | 1156 | 173 | 999.7 | 31.1 | 1.2 | 10.2 |
| **Niu-Xi (NX)** | 牛膝 | 1048 | 198 | 1121.4 | 35.6 | 1.0 | 8.3 |
| **Huang-Qin (HQin)** | 黃芩 | 1012 | 163 | 970.7 | 29.3 | 1.0 | 9.4 |
| **Xu-Duan (XD)** | 續斷 | 1009 | 170 | 948.3 | 30.6 | 1.1 | 8.7 |
| **Da-Huang (DH)** | 大黃 | 1008 | 121 | 645.0 | 21.8 | 0.8 | 7.7 |
| **Du-Zhong (DZ)** | 杜仲 | 976 | 164 | 932.7 | 29.5 | 1.1 | 8.5 |
| **Gu-Sui-Bu (GSB)** | 骨碎補 | 867 | 133 | 727.2 | 23.9 | 1.1 | 8.8 |
| **Bei-Mu (BM)** | 貝母 | 823 | 147 | 865.2 | 26.4 | 1.3 | 8.5 |
| **Mai-Men-Dong (MMD)** | 麥門冬 | 789 | 165 | 996.6 | 29.7 | 1.1 | 7.9 |
| *Sorted by frequency of prescriptions. | | | | | | | |
| Information are obtained from the websites (http://www.americandragon.com/index.htm; http://old.tcmwiki.com/; http://www.shen-nong.com/eng/front/index.html; http://www.ipni.org/; http://www.theplantlist.org/). | | | | | | | |

| **TABLE S2 \|** Composition of the most commonly used herbal formulas and single herbs for hip fracture patients in Taiwan. | | | |
| --- | --- | --- | --- |
| **Formulas** | **Chinese name** | **Number of herbs** | **Composition (Pin-yin name (latin name; botanical plant name))** |
| **Total** |  |  |  |
| **Herbal formula (Pin-yin name)** |  |  |  |
| **Shu-Jing-Huo-Xue-Tang (SJHXT)** | 舒經活血湯 | 17 | **Dang-Gui** (*Radix Angelicae Sinensi*; *Angelica sinensis (Oliv.) Diels*)**, Bai-Shao** (*Radix Paeoniae Alba*; *Paeonia lactiflora Pall.*), **Chuan-Xiong** (*Rhizoma Chuanxiong*; *Ligusticum sinense Oliv.*), **Di-Huang** (*Radix Rehmanniae*; *Rehmannia glutinosa (Gaertn.) DC.*), **Tao-Ren** (*Semen Persicae*; *Prunus persica (L.) Batsch*), **Bai-Zhu** (*Rhizoma Atractylodis*; *Atractylodes macrocephala Koidz.*), **Fu-Ling** (*Poria*; *Wolfiporia extensa (Peck) Ginns*), **Niu-Xi** (*Radix Achyranthis Bidentatae*; *Achyranthes bidentata Blume*), **Wei-Ling-Xian** (*Radix Clematidis*; *Clematis chinensis Osbeck*), **Han-Fang-Ji** (*Radix Stephaniae Tetrandrae*; *Stephania tetrandra S.Moore*), **Qiang-Huo** (*Rhizoma seu Radix Notopterygii*; *Notopterygium forbesii var. oviforme (Shan) H.T. Chang*), **Fang-Feng** (*Radix Saposhnikoviae*; *Saposhnikovia divaricata (Turcz.) Schischk.*), **Long-Dan-Cao** (*Radix Gentianae*; *Gentiana lutea L.*), **Bai-Zhi** (*Radix Angelicae Dahuricae*; *Angelica dahurica (Hoffm.) Benth. & Hook.f. ex Franch. & Sav.*), **Chen-Pi** (*Pericarpium Citri Reticulatae*; *Citrus reticulata Blanco*), **Gan-Cao** (*Radix Glycyrrhizae Preparata*; *Glycyrrhiza uralensis Fisch.*), **Sheng-Jiang** (*Rhizoma Zingiberis Recens*; *Zingiber officinale Roscoe*) |
| **Du-Huo-Ji-Sheng-Tang (DHJST)** | 獨活寄生湯 | 15 | **Bai-Zhi** (*Radix Angelicae Dahuricae*; *Angelica dahurica (Hoffm.) Benth. & Hook.f. ex Franch. & Sav.*), **Xi-Xin** (*Herba cum Radix Asari*; *Asarum sieboldii Miq.*), **Fang-Feng** (*Radix Saposhnikoviae*; *Saposhnikovia divaricata (Turcz.) Schischk.*), **Qin-Jiao** (*Radix Gentianae Macrophyllae*; *Gentiana crassicaulis Duthie ex Burkill*), **Sang-Ji-Sheng** (*Herba Taxilli*; *Taxillus chinensis (DC.) Danser*), **Du-Zhong** (*Eucommiae cortex*; *Eucommia ulmoides Oliv.*), **Niu-Xi** (*Radix Achyranthis Bidentatae*; *Achyranthes bidentata Blume*), **Rou-Gui** (*Cinnamomi cortex*; *Cinnamomum cassia (L.) J.Presl*), **Bai-Zhi** (*Radix Angelicae Dahuricae*; *Angelica dahurica (Hoffm.) Benth. & Hook.f. ex Franch. & Sav.*), **Chuan-Xiong** (*Rhizoma Chuanxiong*; *Ligusticum sinense Oliv.*), **Di-Huang** (*Radix Rehmanniae*; *Rehmannia glutinosa (Gaertn.) DC.*), **Bai-Shao** (*Radix Paeoniae Alba*; *Paeonia lactiflora Pall.*), **Ren-Shen** (*Radix Ginseng*; *Panax ginseng C.A.Mey.*), **Fu-Ling** (*Poria*; *Wolfiporia extensa (Peck) Ginns*), **Gan-Cao** (*Radix Glycyrrhizae Preparata*; *Glycyrrhiza uralensis Fisch.*) |
| **Ma-Zi-Ren-Wan (MZRW)** | 麻子仁丸 | 6 | **Huo-Ma-Ren** (*Semen Cannabis*; *Cannabis sativa L.*), **Xing-Ren** (*Semen Armeniacae*; *Prunus armeniaca L.*), **Bai-Shao (***Radix Paeoniae Alba; Paeonia lactiflora Pall.***), Zhi-Shi** (*Fructus Aurantii Immaturus*; *Citrus aurantium L.*), **Hou-Po** (*Cortex Magnoliae Officinalis*; *Hedyotis diffusa Willd.*), **Da-Huang** (*Radix et Rhizoma Rhei*; *Rheum palmatum L.*) |
| **Ji-Sheng-Shen-Qi-Wan (JSSQW)** | 濟生腎氣丸 | 10 | **Shu-Di-Huang** (*Radix Rehmanniae Preparata*; *Rehmannia glutinosa (Gaertn.) DC.*), **Shan-Zhu-Yu** (*Fructus Corni*; *Cornus officinalis Siebold & Zucc.*), **Shan-Yao** (*Rhizoma Dioscoreae*; *Dioscorea oppositifolia L.*), **Ze-Xie** (*Rhizoma Alismatis*; *Alisma plantago-aquatica L.*), **Bai-Shao** (*Radix Paeoniae Alba*; *Paeonia lactiflora Pall.*), **Mu-Dan-Pi** (*Cortex Moutan*; *Moutan officinalis (L.) Lindl. & Paxton*), **Rou-Gui** (*Cinnamomi cortex*; *Cinnamomum cassia (L.) J.Presl*), **Zhi-Fu-Zi** (*Radix Aconiti Lateralis Preparata*; *Astragalus membranaceus (Fisch.) Bunge*), **Chuan-Niu-Xi** (*Radix Cyathulae*; *Achyranthes bidentata Blume*), **Che-Qian-Zi** (*Semen Plantaginis*; *Plantago depressa Willd.*) |
| **Zhi-Gan-Cao-Tang (ZGCT)** | 炙甘草湯 | 11 | **Gan-Cao** (*Radix Glycyrrhizae Preparata*; *Glycyrrhiza uralensis Fisch.*), **Ren-Shen** (*Radix Ginseng*; *Panax ginseng C.A.Mey.*), **Dan-Shen** (*Radix Salviae Miltiorrhizae*; *Salvia miltiorrhiza Bunge*), **Gui-Zhi** (*Cinnamomi ramulus*; *Cinnamomum cassia (L.) J.Presl*), **Sheng-Di-Huang** (*Radix Rehmanniae*; *Rehmannia glutinosa (Gaertn.) DC.*), **Mai-Men-Dong** (*Radix Ophiopogonis*; *Ophiopogon japonicus (Thunb.) Ker Gawl.*), **E-Jiao** (*Colla Corii Asini*; *Equus asinus L.*), **Huo-Ma-Ren** (*Semen Cannabis*; *Cannabis sativa L.*), **Sheng-Jiang** (*Rhizoma Zingiberis Recens*; *Zingiber officinale Roscoe*), **Da-Zao** (*Fructus Jujube*; *Ziziphus jujuba Mill.*), White Wine |
| **Gan-Lu-Yin (GLY)** | 甘露飲 | 10 | **Di-Huang** (*Radix Rehmanniae*; *Rehmannia glutinosa (Gaertn.) DC.*), **Shu-Di-Huang** (*Radix Rehmanniae Preparata*; *Rehmannia glutinosa (Gaertn.) DC.*), **Shi-Hu** (*Herba Dendrobii*; *Dendrobium moniliforme (L.) Sw.*), **Tian-Men-Dong** (*Radix Asparagi*; *Asparagus cochinchinensis (Lour.) Merr.*), **Mai-Men-Dong** (*Radix Ophiopogonis*; *Ophiopogon japonicus (Thunb.) Ker Gawl.*), **Huang-Qin** (*Radix Scutellariae*; *Scutellaria baicalensis Georgi*), **Yin-Chen-Hao** (*Herba Artemisiae Scopariae*; *Artemisia capillaris Thunb.*), **Zhi-Shi** (*Fructus Aurantii Immaturus*; *Citrus aurantium L.*), **Pi-Pa-Ye** (*Folium Eriobotryae*; *Eriobotrya japonica (Thunb.) Lindl.*), **Gan-Cao** (*Radix Glycyrrhizae Preparata*; *Glycyrrhiza uralensis Fisch.*) |
| **Shao-Yao-Gan-Cao-Tang (SYGCT)** | 芍藥甘草湯 | 2 | **Bai-Shao** (Radix Paeoniae Alba; Paeonia lactiflora Pall.), **Gan-Cao** (Radix Glycyrrhizae Preparata; Glycyrrhiza uralensis Fisch.) |
| **Ban-Xia-Xie-Xin-Tang (BXXXT)** | 半夏瀉心湯 | 7 | **Zhi-Ban-Xia** (*Pinellia Rhizome*; *Pinellia ternata (Thunb.) Makino*), **Gan-Jiang** (*Rhizoma Zingiberis Recens*; *Zingiber officinale Roscoe*), **Huang-Qin** (*Radix Scutellariae*; *Scutellaria baicalensis Georgi*), **Huang-Lian** (*Rhizoma Coptidis*; *Coptis chinensis Franch.*), **Ren-Shen** ( Radix Ginseng; Panax ginseng C.A.Mey.), **Da-Zao** (Fructus Jujube; Ziziphus jujuba Mill.), **Zhi-Gan-Cao (** *Radix Glycyrrhizae Preparata; Glycyrrhiza uralensis Fisch***.)** |
| **Xue-Fu-Zhu-Yu-Tang (XFZYT)** | 血府逐瘀湯 | 11 | **Tao-Ren** (*Semen Persicae*; *Prunus persica (L.) Batsch*), **Hong-Hua** (*Flos Carthami*; *Carthamus tinctorius L.*), **Dang-Gui** (*Radix Angelicae Sinensi*; *Angelica sinensis (Oliv.) Diels*), **Chuan-Xiong** (*Rhizoma Chuanxiong*; *Ligusticum sinense Oliv.*), **Chi-Shao** (Radix Paeoniae Rubra; *Paeonia lactiflora Pall.*), **Chuan-Niu-Xi** (*Radix Cyathulae*; *Achyranthes bidentata Blume*), **Chai-Hu** (*Radix Bupleuri*; *Bupleurum falcatum L.*), **Jie-Geng** (*Radix Platycodi*; *Platycodon grandiflorus (Jacq.) A.DC.*), **Zhi-Shi** (*Fructus Aurantii Immaturus*; *Citrus aurantium L.*), **Sheng-Di-Huang** (*Radix Rehmanniae*; *Rehmannia glutinosa (Gaertn.) DC.*), **Gan-Cao** (Radix Glycyrrhizae Preparata; Glycyrrhiza uralensis Fisch.) |
| **Tian-Wang-Bu-Xin-Dan (TWBXD)** | 天王補心丹 | 14 | **Sheng-Di-Huang** (*Radix Rehmanniae*; *Rehmannia glutinosa (Gaertn.) DC.*), **Ren-Shen** (*Radix Ginseng; Panax ginseng C.A.Mey.*), **Tian-Men-Dong** (*Radix Asparagi*; *Asparagus cochinchinensis (Lour.) Merr.*), **Mai-Men-Dong** (*Radix Ophiopogonis*; *Ophiopogon japonicus (Thunb.) Ker Gawl.*), **Xuan-Shen** (*Radix Scrophulariae*; *Scrophularia microdonta Franch*), **Dan-Shen** (*Radix Salviae Miltiorrhizae*; *Salvia miltiorrhiza Bunge*), **Fu-Ling** (Poria; Wolfiporia extensa (Peck) Ginns), **Yuan-Zhi** (*Radix Polygalae*; *Polygala tenuifolia Willd.*), **Dang-Gui** (*Radix Angelicae Sinensi*; *Angelica sinensis (Oliv.) Diels*), **Wu-Wei-Zi** (*Fructus Schisandrae*; *Schisandra sphenanthera Rehder & E.H.Wilson*), **Bai-Zi-Ren** (*Semen Platycladi*; *Platycladus orientalis (L.) Franco*), **Suan-Zao-Ren** (Semen Zizyphi Spinosae; Ziziphus jujuba Mill.), **Jie-Geng** (Radix Platycodi; Platycodon grandiflorus (Jacq.) A.DC.), **Zhu-Sha (**Cinnabaris) |
| **Single herbs (Pin-yin name)** |  |  |  |
| **Yan-Hu-Suo (YHS)** | 延胡索 | 1 | **Yan-Hu-Suo** (*Rhizoma Corydalis*; *Corydalis yanhusuo (Y.H.Chou & Chun C.Hsu) W.T.Wang ex Z.Y.Su & C.Y.Wu*) |
| **Dan-Shen (DS)** | 丹參 | 1 | **Dan-Shen** (*Radix Salviae Miltiorrhizae*; *Salvia miltiorrhiza Bunge*) |
| **Niu-Xi (NX)** | 牛膝 | 1 | **Niu-Xi** (*Radix Achyranthis Bidentatae*; *Achyranthes bidentata Blume*) |
| **Huang-Qin (HQin)** | 黃芩 | 1 | **Huang-Qin** (*Radix Scutellariae*; *Scutellaria baicalensis Georgi*) |
| **Xu-Duan (XD)** | 續斷 | 1 | **Xu-Duan** (*Radix Dipsaci*; *Dipsacus asperoides C.Y.Cheng & T.M.Ai*) |
| **Da-Huang (DH)** | 大黃 | 1 | **Da-Huang** (*Radix et Rhizoma Rhei*; *Rheum palmatum L.*) |
| **Du-Zhong (DZ)** | 杜仲 | 1 | **Du-Zhong** (*Eucommiae cortex*; *Eucommia ulmoides Oliv.*) |
| **Gu-Sui-Bu (GSB)** | 骨碎補 | 1 | **Gu-Sui-Bu** (*Rhizoma Drynariae; Drynaria fortunei (Kunze ex Mett.) J.Sm.*) |
| **Bei-Mu (BM)** | 貝母 | 1 | **Bei-Mu** (*Bulbus Fritillariae Cirrhosae*; *Fritillaria cirrhosa D.Don*) |
| **Mai-Men-Dong (MMD)** | 麥門冬 | 1 | **Mai-Men-Dong** (*Radix Ophiopogonis*; *Ophiopogon japonicus (Thunb.) Ker Gawl.*) |
| *Sorted by frequency of prescriptions. | | | |
| Information are obtained from the websites (http://www.americandragon.com/index.htm; http://old.tcmwiki.com/; http://www.shen-nong.com/eng/front/index.html; http://www.ipni.org/; http://www.theplantlist.org/). | | | |

| **TABLE S3 \|** Cumulative overall mortality rate of hip fracture patients stratified by age and follow-up time (within 365 days after the index date). | | | | | |
| --- | --- | --- | --- | --- | --- |
|  | **Number of Death (N)** | **Cumulative overall mortality rate (%)** |  | **Number of Death (N)** | **Cumulative overall mortality rate (%)** |
| **Patients at < 60 years old** | **CHM users (N = 82)** | |  | **Non-CHM users (N = 72)** | |
| **Follow-up time (day)** |  |  |  |  |  |
| **< 30 day** | 0 | 0.0% |  | 0 | 0.0% |
| **30-60 day** | 0 | 0.0% |  | 1 | 1.4% |
| **60-90 day** | 0 | 0.0% |  | 1 | 1.4% |
| **90-120 day** | 0 | 0.0% |  | 4 | 5.6% |
| **120-150 day** | 0 | 0.0% |  | 4 | 5.6% |
| **150-180 day** | 1 | 1.2% |  | 4 | 5.6% |
| **180-210 day** | 1 | 1.2% |  | 5 | 6.9% |
| **210-240 day** | 2 | 2.4% |  | 5 | 6.9% |
| **240-270 day** | 2 | 2.4% |  | 6 | 8.3% |
| **270-300 day** | 2 | 2.4% |  | 6 | 8.3% |
| **300-330 day** | 2 | 2.4% |  | 8 | 11.1% |
| **330-365 day** | 5 | 6.1% |  | 10 | 13.9% |
|  |  |  |  |  |  |
| **Patients at ≧ 60 years old** | **CHM users (N = 474)** | |  | **Non-CHM users (N = 484)** | |
| **Follow-up time (day)** |  |  |  |  |  |
| **< 30 day** | 2 | 0.4% |  | 5 | 1.0% |
| **30-60 day** | 7 | 1.5% |  | 12 | 2.5% |
| **60-90 day** | 8 | 1.7% |  | 17 | 3.5% |
| **90-120 day** | 11 | 2.3% |  | 28 | 5.8% |
| **120-150 day** | 17 | 3.6% |  | 38 | 7.9% |
| **150-180 day** | 21 | 4.4% |  | 47 | 9.7% |
| **180-210 day** | 22 | 4.6% |  | 55 | 11.4% |
| **210-240 day** | 24 | 5.1% |  | 62 | 12.8% |
| **240-270 day** | 28 | 5.9% |  | 72 | 14.9% |
| **270-300 day** | 31 | 6.5% |  | 78 | 16.1% |
| **300-330 day** | 37 | 7.8% |  | 85 | 17.6% |
| **330-365 day** | 49 | 10.3% |  | 96 | 19.8% |
|  |  |  |  |  |  |
| N, number; CHM, Chinese herbal medicine. | | | | | |
| Cumulative overall mortality rate (%) was calculated as (number of death)/(total number of CHM or non-CHM users) x 100%. | | | | | |
| The index date of this study was from the day on which the 28 cumulative days of CHM treatment were completed. | | | | | |
| The study endpoint for overall mortality was defined as the date of death, the date of withdrawal from the NHI program, or the date of termination of follow-up (December 31, 2012). | | | | | |
| The cumulative overall mortality rate was calculated within 365 days after the index date. | | | | | |
| Log rank test *p* value (*p*= 0.0962) was used to compare the distribution difference of cumulative overall mortality between CHM and non-CHM users in hip fracture patients < 60 years old. | | | | | |
| Log rank test *p* value (*p*< 0.0001) was used to compare the distribution difference of cumulative overall mortality between CHM and non-CHM users in hip fracture patients ≧ 60 years old. | | | | | |

| **TABLE S4 \|** Cumulative overall mortality rate of hip fracture patients stratified by age and follow-up time (within 12 years after the index date). | | | | | |
| --- | --- | --- | --- | --- | --- |
|  | **Number of Death (N)** | **Cumulative overall mortality rate (%)** |  | **Number of Death (N)** | **Cumulative overall mortality rate (%)** |
| **Patients at < 60 years old** | **CHM users (N = 82)** | |  | **Non-CHM users (N = 72)** | |
| **Follow-up time (year)** |  |  |  |  |  |
| **1 year** | 5 | 6.1% |  | 10 | 13.9% |
| **2 year** | 16 | 19.5% |  | 18 | 25.0% |
| **3 year** | 21 | 25.6% |  | 22 | 30.6% |
| **4 year** | 29 | 35.4% |  | 31 | 43.1% |
| **5 year** | 31 | 37.8% |  | 35 | 48.6% |
| **6 year** | 41 | 50.0% |  | 44 | 61.1% |
| **7 year** | 49 | 59.8% |  | 50 | 69.4% |
| **8 year** | 54 | 65.9% |  | 55 | 76.4% |
| **9 year** | 62 | 75.6% |  | 62 | 86.1% |
| **10 year** | 65 | 79.3% |  | 64 | 88.9% |
| **11 year** | 69 | 84.2% |  | 64 | 88.9% |
| **12 year** | 69 | 84.2% |  | 64 | 88.9% |
|  |  |  |  |  |  |
| **Patients at ≧ 60 years old** | **CHM users (N = 474)** | |  | **Non-CHM users (N = 484)** | |
| **Follow-up time (year)** |  |  |  |  |  |
| **1 year** | 49 | 10.3% |  | 96 | 19.8% |
| **2 year** | 124 | 26.2% |  | 170 | 35.1% |
| **3 year** | 179 | 37.8% |  | 236 | 48.8% |
| **4 year** | 228 | 48.1% |  | 281 | 58.1% |
| **5 year** | 266 | 56.1% |  | 338 | 69.8% |
| **6 year** | 307 | 64.8% |  | 362 | 74.8% |
| **7 year** | 345 | 72.8% |  | 387 | 80.0% |
| **8 year** | 380 | 80.2% |  | 412 | 85.1% |
| **9 year** | 403 | 85.0% |  | 432 | 89.3% |
| **10 year** | 415 | 87.6% |  | 446 | 92.2% |
| **11 year** | 440 | 92.8% |  | 462 | 95.5% |
| **12 year** | 440 | 92.8% |  | 462 | 95.5% |
|  |  |  |  |  |  |
| N, number; CHM, Chinese herbal medicine. | | | | | |
| Cumulative overall mortality rate (%) was calculated as (number of death)/(total number of CHM or non-CHM users) x 100%. | | | | | |
| The index date of this study was from the day on which the 28 cumulative days of CHM treatment were completed. | | | | | |
| The study endpoint for overall mortality was defined as the date of death, the date of withdrawal from the NHI program, or the date of termination of follow-up (December 31, 2012). | | | | | |
| The cumulative overall mortality rate was calculated within 12 years after the index date. | | | | | |
| Log rank test *p* value (*p*= 0.1190) was used to compare the distribution difference of cumulative overall mortality between CHM and non-CHM users in hip fracture patients < 60 years old. | | | | | |
| Log rank test *p* value (*p*= 0.0002) was used to compare the distribution difference of cumulative overall mortality between CHM and non-CHM users in hip fracture patients ≧ 60 years old. | | | | | |

| **TABLE S5 \|** Distribution of the cumulative period of CHM treatment of CHM users among hip fracture patients in this study in Taiwan (within 365 days after the index date). | | |
| --- | --- | --- |
| **Cumulative period of CHM treatment (day)** | **CHM users (N = 556)** | |
|  | **N** | **%** |
| **<60 day** | 158 | 28.42 |
| **60-120 day** | 190 | 34.17 |
| **120-240 day** | 131 | 23.56 |
| **>240 day** | 77 | 13.85 |
| N, number; CHM, Chinese herbal medicine. | | |
| *Non-CHM users were defined as without any usage of CHM during the study period. | | |
| Cumulative period of CHM treatment (day) of CHM treatment was started within 365 days after the index date. | | |
| The index date of this study was from the day on which the 28 cumulative days of CHM treatment were completed. | | |
